# Influence of the prehospital administered dosage of epinephrine on the plasma levels of catecholamines in patients with out-of-hospital cardiac arrest

**DOI:** 10.1016/j.heliyon.2021.e07708

**Published:** 2021-08-05

**Authors:** Jun Nakajima, Yusuke Sawada, Yuta Isshiki, Yumi Ichikawa, Kazunori Fukushima, Yuto Aramaki, Kiyohiro Oshima

**Affiliations:** Department of Emergency Medicine, Gunma University Graduate School of Medicine, 3-39-22 Showa-machi, Maebashi, Gunma 371-8511, Japan

**Keywords:** Epinephrine, Cardiopulmonary arrest, Cardiopulmonary resuscitation

## Abstract

**Aim:**

This study evaluated whether the prehospital administered dosage of epinephrine (Ep) influences the plasma levels of catecholamines in patients with out-of-hospital cardiac arrest (OHCA).

**Methods:**

This was a prospective, observational clinical study. Patients with OHCA transferred to our hospital between July 2014 and July 2017 were analyzed. The plasma levels of catecholamines were measured using blood samples obtained immediately upon arrival at the hospital and before the administration of Ep. Patients were divided into three groups based on the prehospital administered dosage of Ep: no prehospital administration (group Z); 1 mg of Ep (group O); and 2 mg of Ep (group T). The levels of catecholamines, as well as the conditions of resuscitation prior to and after arrival at the hospital were compared between the three groups.

**Results:**

We analyzed 145 patients with OHCA (96, 38, and 11 patients in groups Z, O, and T, respectively). Group T exhibited the highest plasma levels of Ep with a statistically significant difference, however, there were no significant differences in the plasma levels of norepinephrine (Nep), dopamine (DOA) and vasopressin (ADH) among the three groups.

**Conclusion:**

The prehospital administered dosage of Ep influences the plasma levels of Ep; however, it does not contribute to the plasma levels of Nep, DOA and ADH in patients with OHCA.

## 1 Introduction

The efficacy of epinephrine (Ep) in patients with cardiopulmonary arrest (CPA) remains controversial [1, 2, 3, 4]. Although the effectiveness of Ep in this setting has been reported [5, 6, 7], these studies did not address critical outcomes, such as survival to discharge and survival to discharge with good neurologic outcome. Perkins et al. performed a randomized controlled trial (RCT) of Ep in adult patients with out-of-hospital cardiac arrest (OHCA) and reported that the use of Ep resulted in a significantly higher rate of 30-day survival than placebo. However, there was no significant difference in the rate of a favorable neurologic outcome between the Ep administered group and placebo [3]. The 2020 International Consensus on Cardiopulmonary Resuscitation (CPR) and Emergency Cardiovascular Care Science With Treatment Recommendations strongly recommend the administration of Ep during CPR with low-to-moderate certainty of evidence [8, 9].

Nevertheless, there are few reports regarding the plasma levels of Ep in severe conditions. Tárnoky et al. reported that the levels of catecholamines, including Ep and norepinephrine (Nep), in the plasma were significantly higher in the non-surviving animals in hemorrhagic shock models [10]. Lindner et al. evaluated the plasma Ep levels in patients with OHCA and showed that, prior to the administration of Ep, they were significantly lower in resuscitated patients than non-resuscitated patients [11]. In addition, we previously reported that the plasma Ep levels prior to the administration of Ep were significantly lower in the ROSC (+) group than the ROSC (−) group in patients with cardiogenic CPA [12].

Prehospital administration of Ep has been widely performed in patients with OHCA. However, its efficacy, including the influence of Ep concentration in the plasma, has not been fully evaluated. We hypothesized that higher dosages of Ep administered in prehospital situations would result in higher Ep levels in the plasma in CPA patients. The purpose of this study was to evaluate whether the prehospital administered dosage of Ep influences the plasma levels of catecholamines.

## 2 Methods

This prospective, observational clinical study included patients with OHCA admitted between July 2014 and July 2017 for whom blood samples were obtained immediately upon arrival at the hospital and before the administration of Ep in the hospital. This study was approved by the ethics committee of our university hospital (IRB #14-13). Written informed consent was provided by relatives of patients with CPA at the time of admission to our university hospital.

Emergency medical services (EMS) in Japan basically performed CPR to all OHCA patients and they transfer those patients to hospitals when an emergency call is carried out except for the situation which is impossible to resuscitate such as crush injury of head, torso transection and mummification. CPR by EMS at the scene was required for all patients. CPR was performed in conformity with the resuscitation guideline established in 2015 by the Japan Resuscitation Council [13]. Blood sample collection, including for ordinary blood tests, was performed immediately upon arrival at the hospital and before the administration of Ep with continuing CPR in the hospital. The administration of Ep in the hospital was performed immediately after blood sampling, according to this guideline.

Blood samples were centrifuged and the plasma was stored at −80 °C. The levels of catecholamines (i.e., Ep, Nep, and dopamine [DOA]), as well as those of vasopressin (antidiuretic hormone [ADH]) in the plasma were measured using high-performance liquid chromatography and radioimmunoassay, respectively (Bio Medical Laboratories Inc., Tokyo, Japan).

Successful resuscitation, ROSC (+), was defined as detection of a pulse at the carotid artery, femoral artery, or radial artery under advanced CPR, and subsequent maintenance of systolic pressure ≥80 mmHg for 1 h with or without continuous administration of vasoconstrictive agents intravenously or intra-osseously [12]. Patients not achieving the above criteria were defined as ROSC (−).

Patients aged <18 years were excluded. In addition, patients without sufficient data on the levels of catecholamines and ADH in the plasma were also excluded. Eligible patients were divided into three groups based on the prehospital administered dosage of Ep: no prehospital administration of Ep (group Z); 1 mg of Ep (group O); and 2 mg of Ep (group T). Not all paramedics are able to do Ep administration in Japan. A qualification is necessary for paramedics to administer Ep to patients in prehospital situation. In addition, a paramedic who has a qualification of Ep administration must obtain permission from a physician by telephone. Therefore, Ep administration as the initiation of advanced life support by the EMS were sometimes impossible for a reason described above. In prehospital situations, it was attempted to administer 1 mg of Ep every 3–5 min during resuscitation for patients in groups O and T. The levels of the catecholamines and ADH, as well as the conditions of resuscitation prior to and after arrival at the hospital were compared between these three groups. The causes of cardiac arrest were decided based on the situations at the time of patients being found, patients' comorbidities (if we could know), and results of blood examinations and imaging studies such as ultrasonography and computed tomography (CT). CTs were performed in all CPA patients of this study (CTs were done after CPR in patients without ROSC). All CT images were interpreted by radiologists in our hospital. When there were no remarkable abnormalities which were considered to be the cause of cardiac arrest in those factors, we considered the cause of cardiac arrest to be cardiac disease. Therefore, speculations are included in ‘cardiac disease’.

### 2.1 Statistical analysis

Descriptive statistics include medians and interquartile range for continuous variables, and counts, numbers, and percentages for categorical variables. Comparisons of categorical variables among the three groups were performed using the chi-squared test or Fisher's exact test. Comparisons of continuous variables between the two groups were done using the Mann-Whitney U test, and among the three groups were performed using the Kruskal–Wallis test, followed by post-hoc analyses to assess the differences between the three groups. The IBM SPSS Statistics version 25.0 software (IBM Japan, Tokyo, Japan) was used for statistical analysis. A p-value <0.05 denoted statistical significance.

## 3 Results

**Figure 1:**
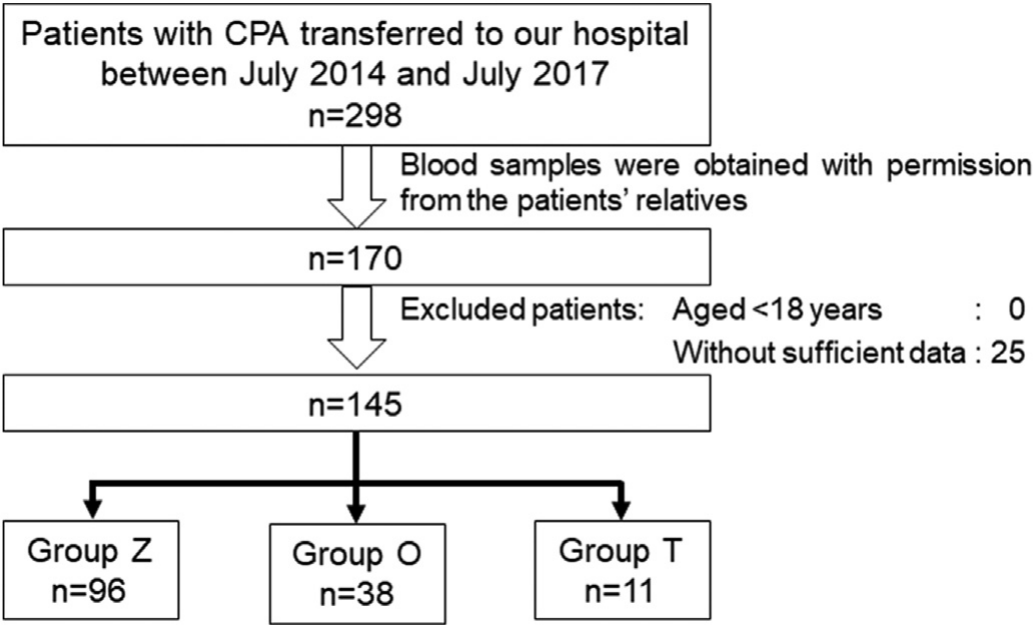
Study flow chart. The analysis included 145 patients with OHCA. OHCA, out-of-hospital cardiac arrest.

A total of 298 patients with OHCA were transferred to our hospital between July 2014 and July 2017. Blood samples were obtained from 170 patients, as described in the Methods section. Twenty-five patients were excluded based on the exclusion criteria. Finally, we analyzed 145 patients in this study. There were 96, 38, and 11 patients in groups Z, O, and T, respectively (Figure 1). Blood samples were obtained before ROSC in all 145 patients.

There were no significant differences in age, male/female ratio, bystander CPR, and electrocardiogram wave form at arrival of EMS among the three groups (Table 1). In addition, there were no significant differences in the duration between the emergency call for an ambulance and EMS arrival, as well as the duration from the last administration of Ep to blood collection (comparison between group O and T), the prehospital and total resuscitation time and the duration between the emergency call for an ambulance and ROSC. Nonetheless, there was a significant difference in the total administered dosages of Ep among the three groups (Table 1).

**Table 1:** Comparisons among three groups.

	Group Z (n = 96)	Group O (n = 38)	Group T (n = 11)	p-value
Age (years)	81 (69, 86)	79 (63, 86)	74 (64, 87)	0.450
Male/female ratio	53/43	24/14	8/3	0.430
Bystander CPR (+)	50.0% (48/96)	31.6% (12/38)	54.5% (6/11)	0.128
ECG at EMS arrival				0.256
VF	8	3	2	
PEA	28	5	2	
Asystole	60	30	7	
Duration between the emergency call for an ambulance and EMS arrival (min)	6.0 (5.0, 7.3)	7.0 (5.0, 8.0)	7.0 (6.0, 9.0)	0.209
Prehospital resuscitation time (min)	18.0 (14.0, 22.0)	19.0 (14.3, 22.0)	17.0 (12.5, 29.5)	0.677
Duration from the last administration of Ep to blood collection (min)	-	3.0 [2.0, 3.0]	3.0 [2.5, 3.5]	0.610
Total administered dosages of Ep (mg)	3.0 (1.0, 5.0)	4.0 (3.0, 5.0)	6.0 (5.0, 6.5)	<0.001∗
Total resuscitation time (min)	36.5 (27.0, 47.5)	38.0 (34.0, 51.0)	43.0 (39.0, 50.5)	0.139
Duration between the emergency call for an ambulance and ROSC (min)	22.0 (14.3, 24.8) (n = 34)	19.0 (19.0, 25.0) (n = 6)	34.0 (33.0, 35.0) (n = 2)	0.307

Total resuscitation time: time between the start of CPR by bystanders or paramedics and the end of CPR in hospital.

CPR: cardiopulmonary resuscitation, ECG: electrocardiogram, VF: ventricular fibrillation, PEA: pulseless electrical activity, EMS: emergency medical service, Ep: epinephrine, ROSC: return of spontaneous circulation.

Data are shown as counts, numbers or medians (Q1, Q3), ∗p < 0.05.

Table 2 shows the causes of CPA in each group; cardiac disease (including speculations) was dominant in all groups, and there was no significant difference in the causes of CPA among the three groups (p = 0.355).

**Table 2:** Causes of cardiopulmonary arrest.

	Group Z (n = 96)	Group O (n = 38)	Group T (n = 11)	p-value
Causes of cardiopulmonary arrest				0.355
Cardiac disease (including speculations)	49 (51.0%)	24 (63.2%)	5 (45.4%)	
Asphyxia	17 (17.7%)	5 (13.1%)	0	
Respiratory disease	11 (11.5%)	3 (7.9%)	1 (9.1%)	
Aortic dissection	7 (7.3%)	1 (2.6%)	2 (18.2%)	
Intracranial hemorrhage	4 (4.2%)	0	1 (9.1%)	
Trauma	3 (3.1%)	2 (5.3%)	0	
Gastrointestinal bleeding	2 (2.1%)	0	1 (9.1%)	
Aortic rupture	2 (2.1%)	1 (2.6%)	1 (9.1%)	
Sepsis	1 (1.0%)	2 (5.3%)	0	

**Figure 2:**
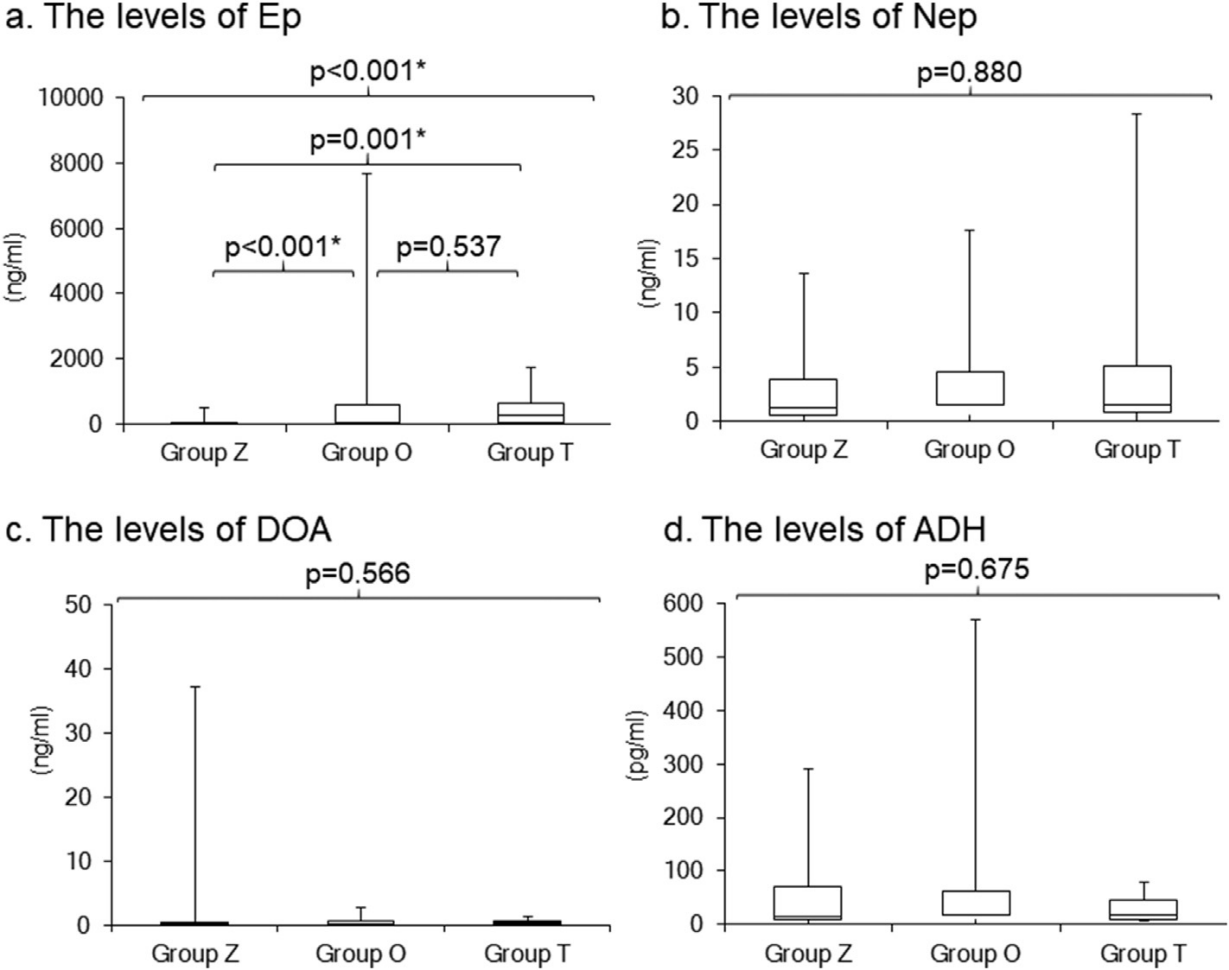
Comparisons of the levels of Ep (a), Nep (b), DOA (c) and ADH (d) in the plasma among the three groups. Ep, epinephrine; Nep, norepinephrine, DOA, dopamine; ADH, antidiuretic hormone. ∗p < 0.05.

Group T exhibited the highest levels of Ep in the plasma, with a significant difference noted among the three groups (normal range: ≤0.10 ng/ml; group Z: 1.95 [0.36, 4.49] ng/ml; group O: 27.65 [5.87, 572.16] ng/ml; and group T: 244.00 [22.10, 620.35] ng/ml; p < 0.001) (Figure 2a). On the other hand, there were no significant differences in the levels of Nep (normal range: 0.10–0.50 ng/ml; group Z: 1.30 [0.63, 3.92] ng/ml; group O: 1.48 [0.54, 4.50] ng/ml; and group T: 1.50 [0.80, 5.15] ng/ml; p = 0.880), DOA (normal range: ≤0.03 ng/ml; group Z: 0.07 [0.02, 0.20] ng/ml; group O: 0.17 [0.01, 0.60] ng/ml; and group T: 0.36 [0.04, 0.53] ng/ml; p = 0.566), and ADH (normal range: ≤4.2 pg/ml; group Z: 14.50 [10.0, 71.10] pg/ml; group O: 18.45 [9.83, 62.00] pg/ml; and group T: 17.50 [10.00, 44.55] pg/ml; p = 0.675) in the plasma (Figure 2b–d).

**Figure 3:**
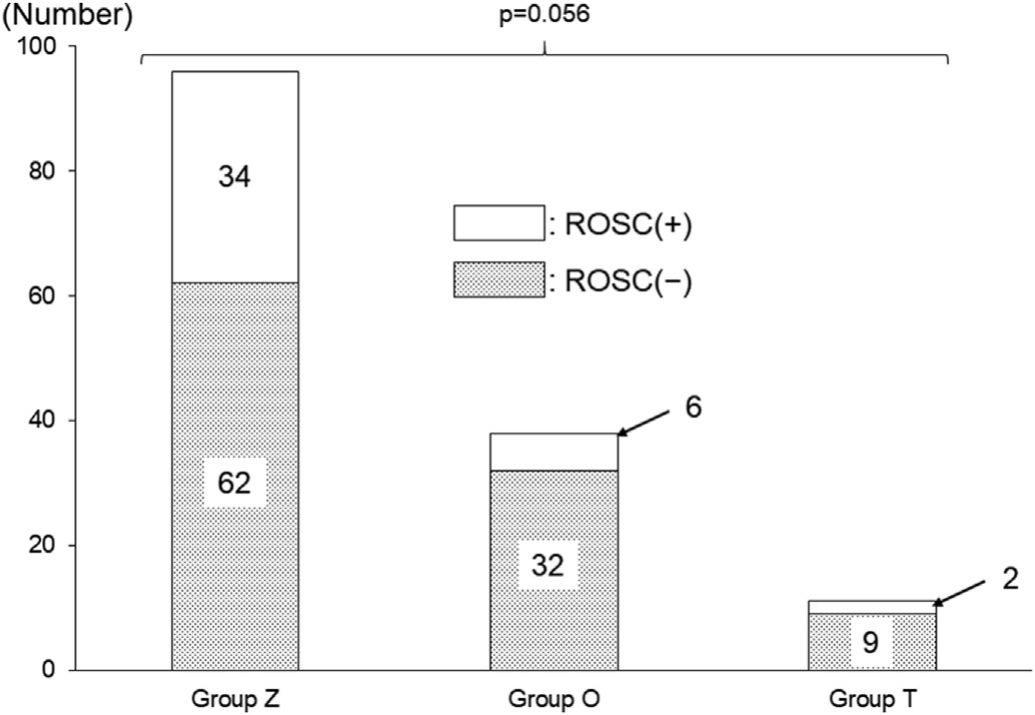
Acquisition of ROSC among the three groups. ROSC, return of spontaneous circulation.

There was no significant difference in the acquisition rate of ROSC among the three groups (35.4%, 15.8%, and 18.2% in groups Z, O, and T, respectively; p = 0.056) (Figure 3).

## 4 Discussion

There is accumulating evidence regarding the role of Ep in CPR. A recent RCT [3] and two reports of systematic review and meta-analysis [14, 15] showed that the administration of Ep did not lead to satisfactory outcomes in the long-term prognosis of patients with OHCA, despite its potential positive effect on short-term prognosis.

Negative aspects of Ep administration in patients with severe conditions have been reported. Tárnoky et al. [10] reported that the levels of catecholamines, such as Ep and Nep, in the plasma were significantly higher in the non-surviving animals in hemorrhagic shock models. In addition, extremely high levels of catecholamines represented the stage of shock irreversibility in those animals. Lindner et al. measured the plasma levels of Ep and Nep in 60 patients with OHCA. They reported that the plasma levels of Ep and Nep prior to the administration of Ep were significantly lower in resuscitated patients than non-resuscitated patients [11]. Our previous study showed that the plasma levels of Ep and Nep prior to the administration of Ep were significantly lower in the ROSC (+) group than the ROSC (−) group in patients with cardiogenic CPA. We concluded that increased levels of Ep in the plasma were not associated with the acquisition of ROSC in patients with cardiogenic CPA and may reflect more severe hypoxic-ischemic insults [12].

The effect of prehospital administration of Ep is controversial. Some investigators have reported the efficacy of early administration of Ep for OHCA [16, 17, 18, 19, 20]. Nevertheless, there are also negative findings regarding the prehospital administration of Ep in patients with OHCA. It has been reported that the administration of Ep prior to arrival at the hospital for the treatment of OHCA did not improve the clinical outcome [21]. Based on a systematic review and meta-analysis, Atiksawedparit et al. reported that prehospital administration of Ep did not improve the overall rates of ROSC, hospital admission, and survival to discharge [22]. Through a systematic review and meta-analysis, Loomba et al. demonstrated that the use of Ep for OHCA prior to arrival at the hospital was associated with a significant increase in the risk of poor neurologic outcome at the time of discharge [23]. In addition, an association between the requirement for Ep administration prior to prehospital ROSC and subsequent rearrest has been identified [24]. In this study, group T showed the highest level of Ep in the plasma with a significant difference, however, there were no significant differences in the levels of Nep, DOA and ADH in the plasma among the three groups. In addition, there was no significant difference in the rate of ROSC acquisition among the three groups. Various differences in backgrounds, including emergency medical system, causes of CPA, and electrocardiogram wave form at the time of Ep administration may influence the findings of studies. We have to carefully consider the results of all studies because Ep is currently the only useful vasopressor for CPR.

DOA is converted to Nep by DOA β-hydroxylase [25]; therefore, DOA is a precursor of Nep and Ep. There was no significant difference in the levels of DOA in the plasma among the three groups examined in this study. The reason for this observation is unclear; the metabolism of DOA, including the activities of DOA β-hydroxylase and dopa decarboxylase which play a role in converting dopa into DOA, may be involved in this effect [12, 25].

Thus far, three RCTs involving >1,500 patients with OHCA have compared ADH versus Ep; however, these studies were published >10 years ago [26, 27, 28]. The combined results of these studies did not show benefits of ADH compared with Ep across all outcomes and initial rhythms. The 2020 International Consensus on CPR and Emergency Cardiovascular Care Science With Treatment Recommendations suggest against the administration of ADH in place of Ep during CPR with weak recommendation and very low-certainty evidence [8, 9]. In this study, there was no significant difference in the plasma levels of ADH among the three groups. Moreover, the increase in the plasma concentration of Ep did not influence the plasma levels of ADH in patients with OHCA.

There were some limitations in this study. This was a prospective study, performed at one institute with a small number of patients. The cause of CPA was not simple and included both endogenous and exogenous etiologies. The disproportion of blood distribution by CPR may indicate that the levels of Ep in the plasma were not proportional to the prehospital doses of Ep. Further studies are warranted to investigate the efficacy of prehospital administration of Ep.

In conclusion, the prehospital administered dosage of Ep influences the plasma levels of Ep, however, it does not contribute to the plasma levels of Nep, DOA and ADH in patients with OHCA.

## Declarations

### Author contribution statement

Jun Nakajima and Kiyohiro Oshima: Conceived and designed the experiments; Performed the experiments; Analyzed and interpreted the data; Contributed reagents, materials, analysis tools or data; Wrote the paper.

Yusuke Sawada: Conceived and designed the experiments; Performed the experiments; Analyzed and interpreted the data; Contributed reagents, materials, analysis tools or data.

Yuta Isshiki: Performed the experiments; Contributed reagents, materials, analysis tools or data.

Yumi Ichikawa, Y, Kazunori Fukushima and Yuto Aramaki: Performed the experiments.

### Funding statement

This research did not receive any specific grant from funding agencies in the public, commercial, or not-for-profit sectors.

### Data availability statement

Data will be made available on request.

### Declaration of interests statement

The authors declare no conflict of interest.

### Additional information

No additional information is available for this paper.
